# Sperm Production Is Reduced after a Heatwave at the Pupal Stage in the Males of the Parasitoid Wasp *Microplitis* *rufiventris* Kok (Hymenoptera; Braconidae)

**DOI:** 10.3390/insects12100862

**Published:** 2021-09-23

**Authors:** Ahmed M. El-Sabrout, Esmat Hegazi, Wedad Khafagi, Christophe Bressac

**Affiliations:** 1 Institut de Recherche sur la Biologie de l’Insecte (IRBI), UMR 7261 CNRS-University of Tours, 37200 Tours, France; elsabroutahmed@alexu.edu.eg or; 2 Applied Entomology and Zoology Department, Faculty of Agriculture (El-Shatby), Alexandria University, Alexandria 21526, Egypt; eshegazi@hotmail.com; 3 Plant Protection Research Institute (PPRI), Alexandria 21526, Egypt; wedademam.khafagi@gmail.com

**Keywords:** heat stress, biocontrol, testis, seminal vesicle, sex-ratio

## Abstract

**Simple Summary:**

Biocontrol with natural enemies of insect pests needs an optimal reproduction of beneficial insects. Most insects are sensitive to heat, and many males suffer from sperm decrease when exposed to warmth during their development. It is dramatic in hymenoptera because males are issued from the development of unfertilized oocytes and only females develop from fertilized eggs. The sex ratio of populations then results from the availability of sperm for egg laying females. *Microplitis* *rufiventris* is a parasite of the cotton worm; this moth is a major pest for cotton fields in Egypt. Because the temperature is high in Egypt, reproduction of *M. rufiventris* must be studied to optimize its use in the fields. We conducted experiments to measure the sperm number of males after heat periods during their development. It shows that *M. rufiventris* males have less sperm than controls when they were exposed to 36 °C and 40 °C short periods during their development. Moreover, those males live shorter than males that were maintained at 25 °C. In conclusion, we found, males to be sensitive to heat waves, which results in lower fertility, resulting in a lower availability of sperm for females leading to a sex ratio bias. It may lead to a decrease of the efficacy of biocontrol in cotton fields.

**Abstract:**

Understanding reproduction is essential for controlling pests and supporting beneficial insects. Among the many factors allowing optimal reproduction, sperm availability is key to sex ratio control in hymenopteran parasitoids since males are haploid and only females come from fertilization. *Microplitis* *rufiventris* (Hymenoptera; Braconidae) is a solitary endoparasitoid of some noctuids. This insect could be used for the control of the cotton leafworm *Spodoptera* *littoralis*. Under controlled conditions, sperm quantity was measured in virgin males at 1, 5, 10, and 15 days; it increases in adult males until the fifth day. Sperm stock of control males increased from 2500 at one day to 6700 at 15 days. With the control climatic condition being 25 °C, we tested the effects of a time-limited increase of temperature that can be found in Egypt (36 and 40 °C) during one day at the early pupal stage. Emerging males had 1500 and 420 sperm at 36 and 40 °C, respectively; both lived shorter than the control. The sperm potential of males is dependent on both age and temperature during the early pupal stage. It could have dramatic consequences on the sex ratio of *M. rufiventris* in natural and controlled populations.

## 1. Introduction

Reducing chemical pesticides is an emergency for human and wildlife health. The concept of “one health” is now well understood, and promotes environmentally friendly agricultural practices. The use of biocontrol agents against pest insects is a strategy that has been used for a long time and continues to be used, but it still needs to be optimized to local agro environments, such as cropping systems, local practices, and climate. Among biocontrol insects, parasitoid wasps (Hymenoptera) are widely used, the necessary condition being that their mass breeding is managed and that the egg-laying females are efficient in the local environment of the release. Those insects are particularly sensitive to climatic conditions because they are at the third and sometimes fourth trophic levels in natural and agronomic ecosystems [1].

Insects are vulnerable to temperature variations because of their small size and ectothermic physiology [2]. Temperature ranges, either cold or warm, suffered by successive developmental stages, play a leading role in their survival and reproductive success [3,4,5]. When temperatures exceed an insect’s optimum temperature range, there are three mutually exclusive results: either survival with accurate physiology, a sublethal effect which decreases some vital functions, or death [6,7]. Among traits currently known to be affected by heat stress, reproductive processes are often diminished by less severe conditions than those causing mortality [5,8,9]. From an ecological and agronomic point of view, sublethal tests are considered more relevant than survival because the temperature variations used are closer to natural conditions [10,11]. The actual temperature is fluctuating in the field, and exposure to high temperature may happen only during short bouts of time in a day, even during heatwave episodes [12,13].

*Microplitisrufiventris* is a braconid Hymenoptera. It parasitizes larvae of some Noctuidae (Lepidoptera) which are agricultural pests. This insect is a promising biological agent for the control of *Spodoptera* *littoralis*, the cotton leafworm [14]. It is a solitary endoparasitoid, meaning that larvae develop inside the host body and only one wasp emerges from its host at the last larval stage, and with koinobiont, the host survives during the complete larval development until the exit of the last instar parasitoid larvae. Afterward, the complete pupal development occurs inside a silk cocoon until adult emergence, and the body size of the insect does not change along this last immature stage.

In all Hymenoptera, due to arrhenotokous parthenogenesis, males are haploid and only females are derived from fertilized oocytes. The sex ratio of the offspring laid by one female is constrained by its sperm stock in the spermatheca after copulation, making sperm availability a key point for population dynamics [15].

In all insects, spermatozoa are produced in the male testis, and the sperm stock is located in seminal vesicles until ejaculation. In males, the constitution of mature sperm stock can occur either or both before the complete development during immature stages, or in the imago (see [16] for a review). In some species, spermatogenesis can be synchronous, all germinal cells being at the same stage in the testes, and males being unable to refill their stock after depletion, as seen in social Hymenoptera [17], and also in non-social parasitoids (*Nasonia*, [18]). On the other hand, spermatogenesis can be continuous, sperm being produced during a prolonged period, allowing rebuilding of the sperm stock after successive copulations of the male, also found in parasitoid wasps from various families [19,20]. Ranking of species from being strictly synchronous with only one wave of spermatogenesis to being fully continuous, gave rise to a spermatogenic index to classify species for this reproductive trait [21].

In most organisms, heat stresses on reproduction are sex-specific [22]. The consequences of heat on males were evidenced in Drosophila, where males are more sensitive than females [23]. It was shown in *Drosophila subobscura* that the fertility of males from cold climates was more affected by heat than those from warmer climates [24]. In the flour beetle *Tribolium* *castaneum*, the sperm production and viability were reduced after time-limited high temperatures suffered by males [25]. It was shown in some parasitoid wasps (Pteromalidae) that the male sperm production was diminished by heat waves during pupal stages and that the sex ratio of the next generation was strongly male-biased [18,26]. Such physiological sensitivity was also observed after chill shocks [27] and consumption of chemicals during larval development [28]. It reveals that egg-laying females were constrained by sperm quantities after mating with these males with decreased sperm. In the context of biological control of agricultural pests by their natural enemies, a decrease of female offspring production due to an insufficient sperm stock has dramatic consequences on sex ratio, and consequently on the success of field applications.

Considering reproductive functions, experimental studies of the consequences of high temperatures on insects lead to three successive thresholds that are from high to low temperatures, that is, lethality (more than 50% do not survive), sublethality (more than 50% survive but could have detrimental handicaps, including sterility), and subfertility, which is decreased fertility of both sexes. The moth *Plutella xylostella* for instance, which is regularly cultured at 25 °C, a 40 °C period during larval or adult stages, induced a diminution of egg production, which was proportional to the time spent in heat [29]. In males of *Anisopteromalus* *calandrae* (Hymenoptera: Pteromalidae), usually cultured at 30 °C, a 72 h heat elevation during the early pupal stage is lethal at 42 °C, sublethal at 40 °C (lower survival and sterile males), and decreases sperm production from 36 °C [26]. In *Nasonia* *vitripennis* (Pteromalidae), a 24 h heat period induced a male subfertility [18]. In most parasitoid wasps, the pupal stage is more sensitive to heat stress because it is immotile, inside the cocoon; the previous stages, which are eggs and larvae inside the host allow them to escape from heat spots. Moreover, it is during the pupal stage that testis begin spermatogenesis, and then become more susceptible to any stress disrupting reproduction [5,16,18].

As yet, a heat sensibility of male fertility in non-Pteromalid parasitoid wasps has not documented. It is a key point in Egypt because this region essentially has a hot desert climate, and temperatures may vary with episodes of high heat over longer or shorter times [30]; episodes of extreme temperature have recently strongly increased. To become a biocontrol agent, the temperature range favoring reproduction in *M. rufiventris* should be determined, especially environmental factors affecting the sex ratio of generations. We conducted an experimental lab study to measure the survival and evolution of sperm supply of males at both control temperature and after different heat waves at the early pupal stage.

## 2. Materials and Methods

Virgin *Microplitis* *rufiventris* females were allowed to lay their eggs in 4th instar larvae of *Spodoptera* *littoralis* fed with cotton leaves. Both were lab strains mass cultured in Alexandria, Egypt. Then, parasitized host larvae were maintained at 25 °C [14] under ambient humidity with food until the emergence of parasitic larvae. Immediately, free wasp larvae weave a silk cocoon. Less than 1 h after the cocoon construction, wasp pupae were individually transferred in plastic vials plugged with cotton and maintained at 25 °C. Some cocoons were opened every 4 h to detect the white pupal stage (the pupae are not colored, and the eyes are orange). This stage of development was identified from both preliminary observations and the description of spermiogenesis in the phylogenetically closely related wasps *Cotesia* *congregata* [31]. Under such controlled conditions, the accurate stage is reached during the 4th day after emergence from the host.

1. Heat survival: Four day old cocoons were transferred to other climate chambers for 24 h at 28, 30, 36, 38, 40 and 42 °C respectively (*n* = 30 to 100 per treatment). Then, they were returned to the original climate chamber at 25 °C until the emergence of adult males. Emerging males were counted in each set to measure survival and sperm count in seminal vesicles (see Section 2.2).
2. Sperm stock enumeration: Males were dissected to count sperm in both seminal vesicles, after DAPI staining (methods in [20]). Briefly, seminal vesicles were isolated with fine forceps and opened in saline solution; sperm were fixed by paraformaldehyde 4%, then by ethanol, and labeled with DAPI to be exhaustively numbered under fluorescence at a 40x objective. Counts of both vesicles were added to obtain the whole sperm stock. Pictures of the tract and spermatozoa were taken under phase contrast and fluorescence to describe the reproductive apparatus.
3. Heat and age consequences on male sperm stock and survival: From the first series, 42 °C being a lethal temperature, and 28 and 30 °C not efficient for sperm decrease, another set of 4 day-old cocoons was maintained at 25 °C (control), or exposed at the white pupal stage for 24 h at either 36 °C and 40 °C. After, series of virgin males were isolated in a vial (plastic microtubes plugged with cotton with a drop of honey) at 25 °C continuously in the dark and dissected for sperm counts after 1 day (25 °C *n* = 13, 36 °C *n* = 13, 40 °C *n* = 13), 5 days (25 °C *n* = 13, 36 °C *n* = 15, 40 °C *n* = 10), 10 days (25 °C *n* = 11, 36 °C *n* = 10, 40 °C *n* = 3) and 15 days (25 °C *n* = 10, 36 °C *n* = 11, 40 °C *n* = 3). Others (*n* = 16 for each treatment) were maintained in isolation under the same conditions and checked twice a day until their death for survival recording.
4. Statistical analysis: Sperm counts were presented as means ± SE. Stats were done with R studio [32]. Sperm counts along ageing and after heat periods were treated as global populations by the Kruskal-Wallis test, and, when significant, followed by Wilcoxon posthoc tests to compare series of males by age and/or temperature. Survivals after heatwaves were compared by a log-rank test.

## 3. Results

**Figure 1:**
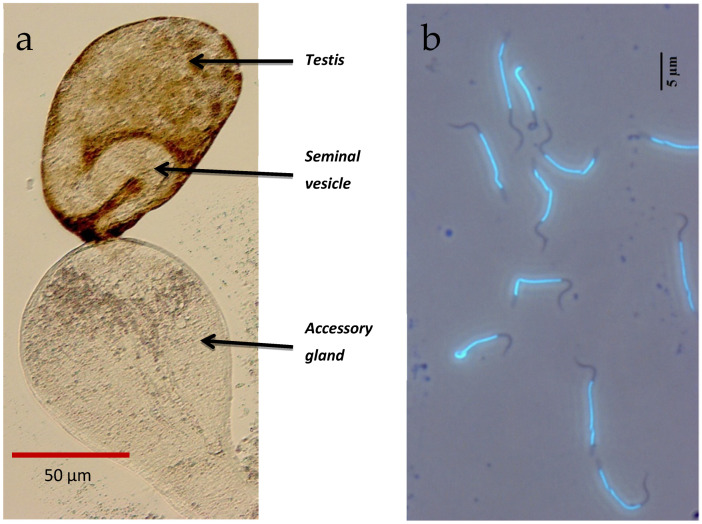
*M. rufiventris* half reproductive tract under phase contrast (**a**); spermatozoa labeled with DAPI (**b**).

The male reproductive tract consists of paired testes and tubular seminal vesicles surrounded by a common conjunctive cap and a pair of accessory glands joining in a common duct (Figure 1).

Spermatozoa are short (ca 15.5 µm, *n* = 8) and the nucleus is longer than the flagellum (Figure 1). They are individualized and not embedded in a mass or a cohesive fluid during storage in seminal vesicles.

**Figure 2:**
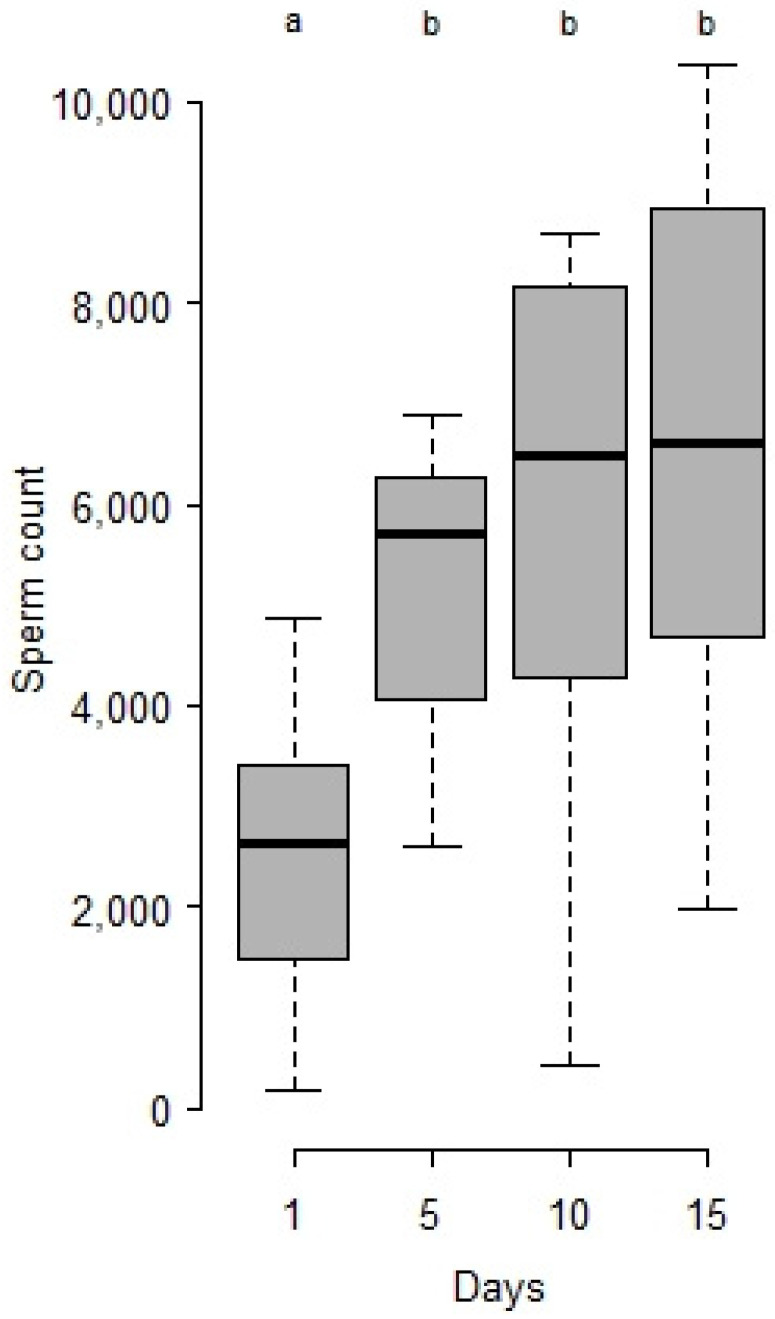
Sperm counted in seminal vesicles of *M. rufiventris* virgin males during the course of aging at 25 °C. Kruskal–Wallis test, Chi^2^ = 19.8, df = 3, *p* < 0.001. Letters indicate series that are statistically different from the previous one (Wilcoxon tests, 1 vs. 5 days, W = 12, *p* < 0.001; 5 vs. 10 days, W = 55, *p* = 0.36; 10 vs. 15 days, W = 45, *p* = 0.51) 200.

Male sperm stock in seminal vesicles increases at the beginning of aging (Figure 2), being 2.1 times more at 5 days than at emergence. It then remains statistically stable up to 15 days (Wilcoxon test, on Figure 2).

**Figure 3:**
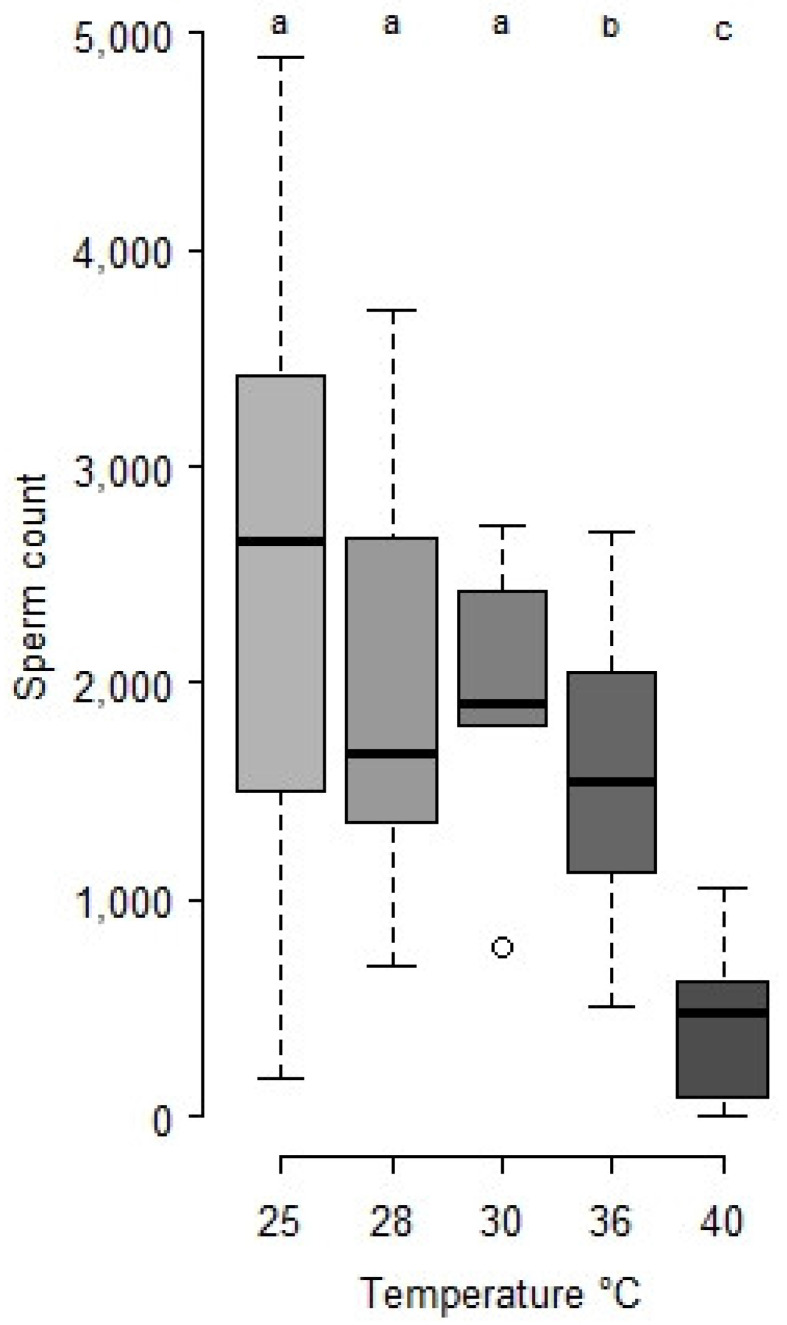
Sperm stock in seminal vesicles of *M. rufiventris* 1-day old virgin males developed at 25 °C after a 24 h heat period at the white pupal stage. The number of males is in the text. Kruskal–Wallis test, Chi^2^ = 30.1, df = 4, *p* < 0.001. Letters indicate statistical differences from the 25 °C control (Wilcoxon test, a for *p* > 0.1, b for *p* < 0.05, c for *p* < 0.001).

The initial sperm stock of virgin males at control temperature is 2467.4 ± 361.8 (*n* = 13), it is not statistically different from males that were exposed to 28 and 30 °C during 24 h at the early pupal stage (Figure 3). However, exposure at 36 and 40 °C produced males that were less fertile than controls, with respectively 1717.7 ± 162.5 (*n* = 14) and 418.2 ± 84.6 (*n* = 14) sperm in their seminal vesicles.

**Figure 4:**
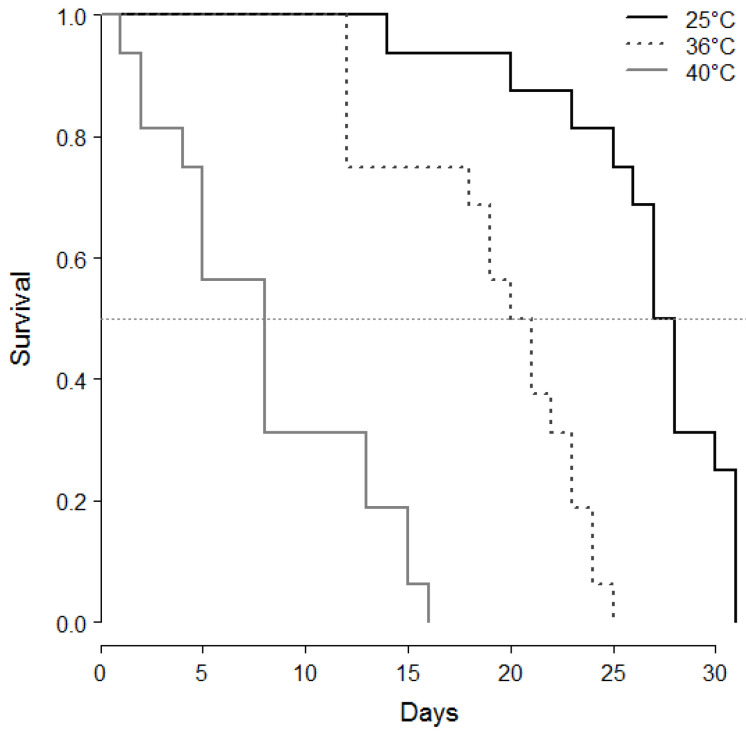
Survival of *M. rufiventris* virgin males fed with honey at 25 °C. Three series are 25 °C—developed continuously at 25 °C (*n* = 39), 36 °C—developed at 25 °C with a 1-day heat period at 36 °C at early pupal stage (*n* = 40), 40 °C—developed at 25 °C with a 1-day heat period at 40 °C at early pupal stage (*n* = 40). Dashed horizontal line indicates 50% of survival. log-rank test, Chi^2^ = 59.4, df = 2, *p* < 0.001.

Male survival is presented in Figure 4. Exposure to high temperature at the pupal stage dramatically diminishes the survival of adult males. Series are statistically different (log-rank test, Chi^2^ = 59.4, df = 2, *p* < 0.001). The mid survival (50%) is reached after 28 days for control males, 21 days for males exposed at 36 °C and only 8 days for males exposed at 40 °C.

**Figure 5:**
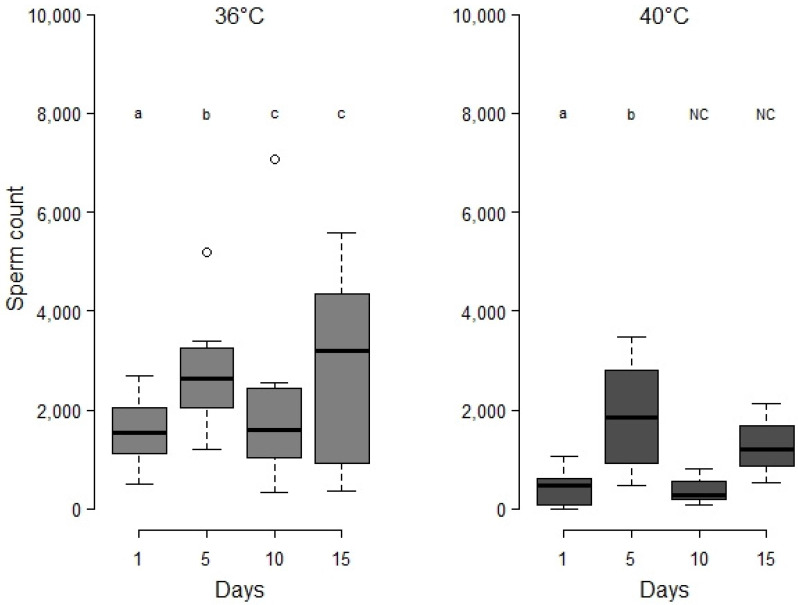
Evolution of *M. rufiventris* male sperm stock in seminal vesicles after a 24 h heat period at white pupal stage. Series of virgin males were considered respectively at 1, 5, 10, and 15 days. Number of males are for 36 °C respectively *n* = 14; 14; 10; 11 and for 40 °C *n* = 14; 10; 3; 3. Kruskal–Wallis tests: for 36 °C, Chi^2^ = 8.8, df = 3, *p* < 0.05; for 40 °C, Chi^2^ = 15.3, df = 3, *p* < 0.01. Letters indicate series that are statistically different from the previous one (Wilcoxon test, *p* < 0.05); for 40 °C, data at 10 and 15 days do not meet Wilcoxon test requirements of *n* > 5 (NC).

Sperm increasing during aging was not visible when males were exposed at 36 °C during 24 h at the white pupal stage. At 40 °C, sub-fertility is amplified (Figure 5). Regardless of the temperature of heat event, there was no visible catching up of sperm stocks during the aging process, and the sperm count at 15 days never reached that of control males. It should be noted that for 40 °C, the periods of 10 and 15 days are longer than the mid survival, which means that only the survivors were taken into account (Figure 5). The preparation for sperm counting does not allow assessment for their viability but sperm are individually observed under the microscope and no obvious aberrations were noticed; their morphology does not seem to differ from controls.

## 4. Discussion

The sperm stock of males of *M. rufiventris* increases at the beginning of their adult life. One-day exposure to high temperature results in subfertility and shorter life duration. Sperm production is sensitive to heat waves during early pupal development, leading to a significant decrease compared to control males, suggesting a response to heat stress. It differs from the continuous warm temperature that gives rise to extra mortality as soon as the temperature reaches 34 °C [14]. This fall in longevity is in line with the results found for the parasitoid wasp *Aphidius* *ervi* (Braconidae) developing in aphid larvae at 15–30 °C [33]. Likewise, in *Aphidius* *colemani* (Braconidae), a heat shock at 34 °C during pupal stage resulted in a reduction of lifespan for both sexes [34].

Both tract and dynamics of sperm production are similar to *Cotesia* *congregata* [31]. Spermatozoa are short, as it is observed in some braconids [35], nuclei being longer than flagella as in *Cotesia* *congregata* and all the microgastrinae subfamily. However, the length of the spermatozoa is not as small as in *C. congregata*, which is known for its extremely short sperm [31]. Sperm are released continuously in seminal vesicles during the first 5 days of adult life, indicating continuous spermatogenesis, with a spermatogenic index close to one, according to [21]. That sperm availability could allow males to mate with successive females and distribute their sperm across time. *Microplitis* males are fully winged and fly well; they may be able to reach females as they emerge. This is different from honeybee drones for instance, which have to transfer their whole sperm reserve in one copulation, mixing it with sperm of other males in the queen’s spermatheca [36].

Total sperm counts (maximum of 6700 after 5 days) are of similar magnitude compared to other parasitoid wasps. It is not a reproductive trait associated uniquely with short sperm because in *Nasonia* *vitripennis* (Pteromalidae), whose bodysize is close to *M. rufiventris*, i.e., 2–3 mm, sperm measure c.a. 150 µm, male produce less than 10,000 sperm and females store about 1,000 sperm [18]. In the extremely short sperm *Cotesia* *congregata*, the maximum sperm production reaches 30,000 and females store 1000 [20]. In all parasitoid wasps investigated for sperm counts, the number of fertilized eggs (i.e., female offspring) is close to the sperm stored in the spermatheca, then sperm have a great paternity expectancy [15].

In *M. rufiventris*, sperm stock is 2500 in one-day old control males vs. 420 in males having undergone a 40 °C heatwave at the pupal stage. Those control males live 28 days, while when heat-stressed lifespan is only 8 days. Consequently, the fitness reduction is not restricted to fertility but also to the opportunity to meet virgin females in a wider area along with a longer life. It was shown in the closely related parasitoid wasp, *Microplitis* *similis*, that the range of optimal temperature for development was between 18 and 33 °C (constant temperatures), and 36 °C prevents any development [37]. Its longevity decreased at 33 °C. Moreover, both male and female fecundities diminished at 33 °C, but the female ratio in the offspring was high, meaning that sperm of males were viable.

The lower male fertility after a heatwave is consistent with other insects as in the pupae of the parasitic wasps *Trichogramma* *brassicae*, Trichogrammatidae [38], or *Drosophila melanogaster* [39]. It can be hypothesized that the diminution of male sperm stock through a heatwave at an immature stage is due to direct injuries to male germlines, leading to the production of rare sperm or empty seminal vesicles [38], inducing a disadvantage for heat-shocked males compared to controls. Moreover, sperm stock after 36 °C heat period does not increase gradually with age as in controls. Sperm dynamics in heat-stressed males appear to change over time, as stocks on successive days are significantly different, but no physiological mechanism, such as putative sperm resorption could explain this variation in parasitoid wasps [5,18,38,40]; we can conclude that this variation in the number of sperm results from interindividual variabilities. In the parasitoid wasp *Dinarmus* *basalis*, males were able to transfer viable sperm to females at the end of their life [40]. In the beetle *Tribolium* *castaneum*, heat waves during immature stages lead to a decrease in male fertility at the level of sperm production, but adults, which could live more than 90 days, recover their full reproduction after 15 days to one month [25]. This lifespan is much longer than *M. rufiventris*. It is clear that *M. rufiventris* share some physiological traits with this Coleoptera considered as a biological model, but many differences in life-history traits make comparisons non-pertinent.

Among parasitoid wasps, heat sensitivity of sperm production was previously evidenced in Pteromalids [5,18,26], and Trichogrammatidae [38], but it is the first observation in Braconids. The state of insect male fertility is not sufficiently known to generalize such physiological consequences of heat stress to all parasitoid wasps. Experimental studies in other taxons are welcome to develop novel comparative analyses of temperature effects on fertility of male parasitoid wasps.

Our study was only conducted on males, and they were not offered females for testing the whole spectrum of fertility. In other parasitoid wasps, a male sperm diminution, either due to heat stress [18,26,27] or to chemical stress [28], has not been accompanied by a decrease of sperm viability, because those rare gametes are fully fertile. However, their low availability in males results successively in a low transfer to females, a low storage in spermathecae, and a resultant sex ratio biased in favor of males. Moreover, heat-stressed males are less competitive than controls of the same age for access to virgin females [26], suggesting that functions other than spermatogenesis were also heat-sensitive as shown in other parasitoid wasps [34].

Such an altered fitness renders heat-stressed males subfertile but not sterile; as a consequence, their presence in populations results in weakness for survival in the environment. This is reinforced by the monoandry tendencies of females, because a vast majority of parasitoid female wasps mate only once [35], even when mated with males having a sperm stock insufficient to fertilize all their eggs [15,41].

The mortality of adults is a measure that is currently recorded in studies of the consequences of stresses of insects. In most experimental works, the sex of insects is not provided, mainly because they are larvae [42], and in others, only females were tested [43]. Here again, comparative data on males are missing. In parasitoids, the thermal sensitivity of hosts is also a major constraint in the face to thermal variations, but it is more effective during the larval development occurring inside the host body [1]. Interestingly, an experimental study on a pest moth showed that larvae, pupae, and adults are sensitive to a heatwave, resulting in a decrease in reproduction in both males and females [29]. This suggests that the larval stage of endoparasitoid may be also subject to physiological damages when their hosts were faced with unfavorable climatic conditions, as seen in *Cotesia* *congregata* during its larval stage inside its host *Manduca sexta* [42]. In koinobiont parasitoids, larvae could be less exposed to unfavorable temperatures because the host can move to find more suitable micro-environments, such as most mobile insects [44]. However, the immobile pupal stage cannot escape from local conditions, and our study helps identify that the pupal stage may be a weak link for this species in the face of exposure to high temperatures. Moreover, the parasitoid and its host may be sensitive to different temperature thresholds, leading to differential survival after thermal stresses [7].

The deleterious consequences of heat stress were shown in the parasitoid *Aphidius* *colemani* wasp [34]. They have varied qualities, such as mortality during development in females being more than in males, and, in adult females, behavioral and physiological disorders. Moreover, it was shown in *Trichogramma* *euproctidis* (Trichogrammatidae) that ovipositing females laid less fertilized eggs at higher temperatures, leading to more male-biased offspring [45]. When these negative effects are accompanied by a sharp drop in male fertility, as it appends in *M. rufiventris*, it forces the rare females to lay unfertile eggs, and this leads to a strong disruption of the natural and released populations of parasitoids.

The perspectives for understanding heat stress in parasitoid wasps could be investigated on both males and females, measuring physiological traits, such as spermatogenesis and oogenesis, and the consequences on their reproduction, particularly on the offspring sex ratio. This would be closer to field conditions with repeated thermal stresses to mimic a daily change in temperature.

When organisms are confronted with unfavorable temperatures during immature stages, the consequences later in life are called ‘carry-over’ effects [46], and are visible as fitness outcomes. Here, we evidenced a strong ‘carry-over’ effect on sperm production. However, it has only negative consequences on fitness, with not only sperm decreasing but the lifespan of males increasing as well. Actually, ‘carry-over’ effects are suspected to reveal phenotypic plasticity with a correlation between the traits, allowing at least maintenance of the fitness [46], or an increase as in the neriid flies where males have bigger secondary sexually traits when they develop with unfavourable diets [47]. Then, consequences of heatwaves in *M. rufiventris* are not good illustrations ‘carry-over’ effects.

## 5. Conclusions

Heat sensitivity of spermatogenesis has dramatic consequences on male fertility, and consequently on the sex ratio of *M. rufiventris* in controlled and natural populations. Such a physiological response to heat stress in unpredictable environments must be considered in natural and cultured populations of parasitoid wasps subjected to high temperatures, especially at the pupal stage. This could be of major interest in the context of global climate change, which includes an increase in the frequency and intensity of heatwave events [9,30]. Moreover, a better knowledge of this temperature sensitivity of males could make it possible to manage genetic selection towards lines that are more resistant in mass cultures to be more efficient when released in the field.

## Data Availability

All data are included in figures, or can be obtained by contacting corresponding author.
